# Evaluation of HPV E6/E7 mRNA in predicting recurrence after Leep in cervical intraepithelial neoplasia: A study of negative findings

**DOI:** 10.1371/journal.pone.0360914

**Published:** 2026-10-16

**Authors:** Yingmei Huang, Jianyong Huang, Hongmian Jiang, Yumei Wei, Zhong Lin, Baoli Xie

**Affiliations:** 1 Gynecology Department, The First People’s Hospital of Nanning, Nanning, Guangxi Zhuang Autonomous Region, China; 2 Pathology Department, The First People’s Hospital of Nanning, Nanning, Guangxi Zhuang Autonomous Region, China; 3 Gynecology Department, The Reproductive Hospital of Guangxi Zhuang Autonomous Region, Nanning, Guangxi Zhuang Autonomous Region, China; Mater Olbia Hospital, ITALY

## Abstract

Cervical intraepithelial neoplasia (CIN) is a precursor to cervical cancer, and early detection of residual or recurrent disease is therefore critical for effective management. This study evaluated whether HPV E6/E7 mRNA status predicts recurrence in patients with CIN who underwent loop electrosurgical excision procedure (LEEP). People’s Hospital of Nanning between January 2019 and July 2022 were included. Patients who underwent LEEP for high-grade cervical lesions at the First Follow-up commenced 4–6 months after LEEP and continued for 24 months; abnormal findings during this period prompted referral for colposcopy or cervical biopsy. Of the 91 patients, 3 (3.30%) experienced recurrence within 24 months. Recurrence rates were similar between patients with preoperative E6/E7 mRNA positivity (3.23%) and negativity (3.45%) (p = 0.956), and between postoperative E6/E7 mRNA status groups (5.26% in the negative group vs. 0% in the positive group; p = 0.814). Although 66.7% of patients shifted from positive to negative E6/E7 mRNA status after LEEP (p < 0.001), this change was not associated with recurrence risk. Preoperative E6/E7 mRNA yielded a sensitivity of 66.67% and specificity of 31.82%, whereas postoperative E6/E7 mRNA showed a sensitivity of 0% and specificity of 97.30%. In conclusion, E6/E7 mRNA status before and after LEEP did not predict recurrence in patients with CIN. Despite a significant reduction in E6/E7 mRNA positivity after surgery, its low sensitivity underscores the need for more effective biomarkers in follow-up care.

## Introduction

Cervical intraepithelial neoplasia (CIN), a common precursor to cervical cancer, is characterized by aberrant cellular alterations in the cervical lining [1–3]. Effective identification of residual or recurrent disease after treatment is therefore crucial for improving patient outcomes, particularly following loop electrosurgical excision procedure (LEEP) [4,5]. Although a number of diagnostic techniques are available, a comprehensive approach combining several tests may improve the sensitivity and specificity of detecting residual disease [6,7].

A “positive margin” after conization has been proposed as a predictor of residual disease; however, studies indicate that nearly half of cases with a positive margin do not progress to residual or recurrent disease, whereas individuals with negative margins may still harbor residual disease [8]. Consequently, margin status alone cannot reliably determine the need for a second LEEP or intensive follow-up.

According to a meta-analysis by Arbyn et al., HPV testing offers higher sensitivity (91% vs. 56%) and comparable specificity (84%) than margin status in predicting residual disease [9]. Although HPV testing has facilitated the detection of residual disease after LEEP, its limited specificity remains a drawback [9].

In recent years, HPV E6/E7 mRNA testing has become increasingly used in cervical cancer screening [10]. This test detects the viral oncogenes E6 and E7, which are responsible for the development and persistence of HPV-related lesions [11]. Owing to its high specificity and its capacity to distinguish between transient and persistent infections, it serves as a useful adjunct to conventional screening methods [12,13]. By identifying the high-risk HPV strains commonly associated with CIN and cervical cancer, the E6/E7 mRNA test enables a targeted assessment of ongoing oncogenic activity and persistent infection [14,15].

Given the increasing use of E6/E7 HPV mRNA testing in the management of HPV-positive women, the high sensitivity and specificity of this method establish it as an effective diagnostic and prognostic biomarker [16–18]. To capitalize on these advantages, we incorporated mRNA testing to monitor recurrence after LEEP. In the present study, we retrospectively analyzed data from women who underwent LEEP for CIN2/3 to evaluate the predictive value of HPV E6/E7 mRNA for recurrence risk following cervical conization.

## Materials and mthods

### Study population and design

We conducted a single-center retrospective cohort study that included patients with high-grade cervical lesions who underwent loop electrosurgical excision procedure (LEEP) at the First People’s Hospital of Nanning between January 2019 and July 2022. Follow-up commenced 4–6 months after surgery and continued for a total of 24 months; abnormal findings during this period prompted referrals for colposcopy or cervical biopsy. This study was reviewed and approved by the Ethics Committee of the First People’s Hospital of Nanning and adhered to the STROBE guidelines. Data were accessed for research purposes on 14 December 2025.

Inclusion criteria were: (1) patients who underwent LEEP for CIN2/3; (2) histologically confirmed negative margins on post-LEEP pathology; (3) availability of preoperative HPV E6/E7 mRNA testing; and (4) documented cervical biopsy evidence for recurrence within two years after LEEP, or completion of a two-year follow-up for non-recurrence cases. Exclusion criteria included: (1) incomplete follow-up; (2) age under 18 years; (3) a positive pregnancy test or a cervical cancer diagnosis after LEEP; and (4) immune, hematologic, hepatic, or renal disorders, or other malignancies.

### Follow-up procedure

Follow-up commenced 4–6 months after surgery, with ThinPrep cytologic testing and HPV screening performed over a two-year period. According to our protocol, HPV-positive women underwent E6/E7 HPV mRNA testing. Women who tested positive for E6/E7 mRNA or who showed abnormalities on ThinPrep cytology were referred for colposcopy. If colposcopy suggested a high-grade cervical lesions, a cervical biopsy was performed. A positive biopsy indicating any histologically confirmed cervical intraepithelial lesion (CIN1+) was defined as recurrence; women with negative results continued regular follow-up

### Leep technique

LEEP was performed by experienced staff under colposcopic guidance and local anesthesia. Loop electrodes 20 mm wide with depths of 12, 15, or 20 mm were selected according to the cervical anatomy and lesion characteristics. Colposcopic examination confirmed complete removal of the lesion, with resection margins extending 2–3 mm beyond the lesion. Histological analysis of the cervical cone assessed margin involvement and provided a definitive diagnosis. The margin was considered positive when the distance between CIN2+ and the resection surface was less than 1 mm.

### HPV E6/E7 mRNA detection

HPV detection was performed using the QuantiVirus HPV E6/E7 mRNA assay (Kodia, Xinxiang, China), which is based on branched-chain DNA hybridization technology. The assay targeted 14 high-risk HPV types: 16, 18, 31, 33, 35, 39, 45, 51, 52, 56, 58, 59, 66, and 68. Hybridization was performed on a microplate, in which viral mRNA formed a branched DNA complex with pre-amplified probes. Detection signals were measured using alkaline phosphatase-labeled luminescent probes and quantified with QuantiVirus luminescence equipment. Results were expressed in relative light units (RLUs) and analyzed using Diacarta software. Signals below the threshold were considered negative, whereas those above the threshold were recorded as positive.

### Statistical analysis

Statistical analyses were performed using SPSS software (version 27.0; SPSS, Chicago, IL, USA). Categorical variables, including baseline characteristics and recurrence-related factors, were compared between groups using the Chi-square test or Fisher’s exact test, as appropriate. Differences in continuous variables between the recurrence and non-recurrence groups were assessed using the Mann-Whitney U test. Changes in preoperative and postoperative E6/E7 mRNA status were evaluated using the McNemar test to determine paired group differences.

To assess the predictive value of preoperative and postoperative E6/E7 mRNA status for recurrence, odds ratios (ORs) with 95% confidence intervals (CIs) were calculated. To quantify the biomarker’s predictive ability, diagnostic performance parameters—including sensitivity, specificity, positive predictive value (PPV), and negative predictive value (NPV) —were also computed. All statistical tests were two-sided, and p-values < 0.05 were considered statistically significant.

## Results

### Baseline characteristics and recurrence rates

A total of 91 patients aged 20–66 years were included. Of these, 3 patients (3.30%) experienced recurrence within 24 months after LEEP. Analysis of baseline characteristics revealed no statistically significant differences in age (p = 0.311) or body mass index (p = 0.396) between the recurrence and non-recurrence groups (Table 1). Preoperative E6/E7 mRNA positivity was observed in 68.1% of patients, whereas postoperative positivity decreased to 1.1%. There was no significant association between preoperative or postoperative E6/E7 mRNA status and recurrence.

**Table 1:** Baseline characteristics of patients with and without recurrence following cervical surgery.

	Participants			
Variables	Total	non-recurrence group	recurrence group	
	(n = 91)	(n = 88)	(n = 3)	P value
Age(year), n (%)				0.311
<30	14 (31.1)	13 (29.5)	1 (100)	
>30	31 (68.9)	31 (70.5)	0 (0)	
BMI(Kg/m^2^), n (%)				0.396
<25	77 (84.6)	75 (85.2)	2 (66.7)	
25-30	11 (12.1)	10 (11.4)	1 (33.3)	
>30	3 (3.3)	3 (3.4)	0 (0)	
Preoperative E6/E7mRNA, n (%)				1
Negativity	29 (31.9)	28 (31.8)	1 (33.3)	
Positivity	62 (68.1)	60 (68.2)	2 (66.7)	
Postoperative E6/E7mRNA, n (%)				0.57
Negativity	38 (41.8)	36 (40.9)	2 (66.7)	
Positivity	1 (1.1)	1 (1.1)	0 (0)	
Postoperative management, n (%)				1
Expectant management	68 (75.6)	65 (74.7)	3 (100)	
Antiviral therapy	21 (23.3)	21 (24.1)	0 (0)	
Post-HPV infection intervention, n (%)				1
Expectant management	73 (80.2)	70 (79.5)	3 (100)	
Medication therapy	8 (8.8)	8 (9.1)	0 (0)	
Physical therapy	1 (1.1)	1 (1.1)	0 (0)	
Number of live births, Median (IQR)	1.0 (0.0, 2.0)	1.0 (0.0, 2.0)	1.0 (0.5, 1.0)	0.375
Number of miscarriages, Median (IQR)	1.0 (0.0, 2.0)	1.0 (0.0, 2.0)	2.0 (1.0, 3.5)	0.33

### Recurrence by preoperative and postoperative E6/E7 mRNA status

Among patients with preoperative E6/E7 mRNA negativity, 3.45% experienced recurrence, compared with a recurrence rate of 3.23% among those with preoperative positivity (p = 0.956) (Table 2). For postoperative E6/E7 mRNA, the recurrence rate was 5.26% in the negative group and 0% in the positive group (p = 0.814), indicating no significant association between postoperative mRNA status and recurrence risk.

**Table 2:** Recurrence according to variables preoperative E6/E7mRNA and postoperative E6/E7mRNA.

Variables	No.	recurrence n (%)	p-value
	91	3(3.30)	
Preoperative E6/E7mRNA			0.956
Negativity	29	1(3.45)	
Positivity	62	2(3.23)	
Postoperative E6/E7mRNA			0.814
Negativity	38	2(5.26)	
Positivity	1	0(0)	

### Changes in E6/E7 mRNA status post-Leep

A significant change in E6/E7 mRNA status was observed after LEEP, with 66.7% of patients shifting from positive to negative (p < 0.001, McNemar test) (Table 3). In addition, 30.8% of patients were negative both preoperatively and postoperatively, whereas 2.6% remained positive throughout.

**Table 3:** Comparison of Preoperative and Postoperative E6/E7mRNA Status.

Preoperative E6/E7mRNA	Postoperative E6/E7mRNA	No.	Percentage (%)	p-value
Negativity	Negativity	12	30.8	
Negativity	Positivity	0	0	
Positivity	Positivity	1	2.6	
Positivity	Negativity	26	66.70	
Total		39	100	<0.001 (McNemar test)

### Predictive value of E6/E7 mRNA for recurrence

The odds ratio (OR) for recurrence based on preoperative E6/E7 mRNA negativity was 1.4 (95% CI: 0.09–23.03, p = 0.813), whereas preoperative positivity yielded an OR of 0.71 (95% CI: 0.04–11.71, p = 0.813). Postoperative E6/E7 mRNA negativity was associated with an OR of 50.27 and a wide confidence interval (95% CI: 0–∞), while postoperative positivity yielded an OR of 0.02 (95% CI: 0–∞), neither was statistically significant (p = 1) (Table 4).

**Table 4:** Odds ratio and p-value by E6/E7 mRNA status post-surgery.

Variables	No.	recurrence n(%)	Odds Ratio (95% CI)	p-value
Overall	91	3(3.30)		
Preoperative E6/E7mRNA				
Negativity	29	1(3.45)	1.4(0.09-23.03)	0.813
Positivity	62	2(3.23)	0.71(0.04-11.71)	0.813
Postoperative E6/E7mRNA				
Negativity	38	2(5.26)	50.27(0, ∞)	1
Positivity	1	0(0)	0.02(0, ∞)	1

### Diagnostic performance of E6/E7 mRNA status

The diagnostic performance of E6/E7 mRNA in predicting recurrence was limited. Preoperative E6/E7 mRNA demonstrated a sensitivity of 66.67% and specificity of 31.82%, with a positive predictive value (PPV) of 3.23% and a negative predictive value (NPV) of 96.55% (Table 5). Postoperative E6/E7 mRNA showed a sensitivity of 0% and a specificity of 97.30%, with a PPV of 0% and an NPV of 94.74%. These results indicate that, although E6/E7 mRNA is highly specific in the postoperative setting, it lacks the sensitivity required to serve as a reliable predictive biomarker for recurrence following LEEP.

**Table 5:** Performance indicators of preoperative E6/E7mRNA and postoperative E6/E7mRNA.

	Sensibility (%)	95% CI	Specificity (%)	95% CI	PPV (%)	95% CI	NPV (%)	95% CI
Preoperative E6/E7mRNA	66.67	20.77–93.85	31.82	23.02–42.13	3.23	0.89–11.02	96.55	82.82–99.39
Postoperative E6/E7mRNA	0.00	0.00–65.76	97.30	86.18–99.52	0.00	0.00–79.35	94.74	82.71–98.54

## Discussion

This study evaluated the predictive utility of HPV E6/E7 mRNA status for recurrence in patients with CIN following LEEP. Despite a significant reduction in mRNA positivity after surgery, neither preoperative nor postoperative E6/E7 mRNA status demonstrated sufficient sensitivity for recurrence prediction. Although preoperative testing exhibited high specificity, its low sensitivity— coupled with the limited predictive capacity of postoperative testing—restricts its value as a standalone biomarker in post-LEEP follow-up. These findings underscore the need for more effective biomarkers to enhance recurrence monitoring and clinical management in patients with CIN.

Long-term research has demonstrated that the risk of cervical cancer can persist for up to 25 years after conization for CIN3, underscoring the necessity of careful monitoring and early disease identification [19]. Experimental studies have shown that HPV E6/E7 mRNA increases the likelihood of high-grade cervical neoplasia by integrating viral DNA into the host genome and causing deletion of the E2 tumor suppressor gene, which regulates the production of E6 and E7 oncogenes [20–22]. Overexpression of E6/E7 is known to drive cervical neoplasia, and E6/E7 mRNA detection is recognized as a direct biomarker of viral carcinogenic activity. Although sensitivity, specificity, PPV, NPV, and AUC values for E6/E7 mRNA range from 65–100%, 42.7–90.2%, 10–85.9%, 66.7–100%, and 0.59–0.80, respectively, variability in participant characteristics affects generalizability [21,23–25].Moreover, diagnostic performance varies widely across studies, with reported specificities ranging from 42.7% [21] to 90.2% [26] and PPVs from 10% [21] to 85.9% [27]. Similarly, AUC values range from 0.59 [23] to 0.80 [28], with differences likely attributable to variations in study populations and cervical pathology.

Previous studies have suggested that HPV E6/E7 mRNA could effectively predict recurrence following LEEP. For instance, Song Y et al.[29] evaluated the diagnostic value of combined HPV E6/E7 mRNA and ThinPrep cytology (TCT) in detecting residual or recurrent cervical disease in 289 cases of high-grade lesion cases after LEEP, finding that combining E6/E7 mRNA with TCT provided higher diagnostic accuracy than either test alone. In contrast, our study assessed E6/E7 mRNA alone and found that it did not effectively predict recurrence as a standalone biomarker. This difference in outcomes may be attributable to the combined approach used in the referenced study, which likely enhanced diagnostic power by addressing both viral oncogene expression and cytological abnormalities. The addition of TCT may also improve sample quality and reveal cytological changes undetectable by mRNA testing alone. These findings suggest that, although E6/E7 mRNA alone may have limited predictive power, it remains valuable when combined with other methods, such as TCT, for comprehensive post-LEEP monitoring.

In addition, M.T. Bruno et al.[30] found HPV E6/E7 mRNA to be an accurate predictive marker of residual disease in a study of 285 high-risk patients with CIN3. The divergent conclusions between that study and ours may result from several factors. That study focused exclusively on patients with CIN3, a high-risk subgroup in which E6/E7 mRNA may be more predictive, whereas our study included both CIN2 and CIN3 cases, potentially diluting its predictive value. Moreover, the longer follow-up in that study allowed residual disease to be detected over a more extended period, whereas the 24-month period in our study may have limited recurrence detection. Variability in mRNA assay sensitivity may also contribute. Taken together, these considerations suggest that, although E6/E7 mRNA may predict residual disease effectively in CIN3, its utility in mixed-grade CIN populations may require additional biomarkers.

Beyond these reports, several other follow-up studies that used E6/E7 mRNA alone also reached conclusions contrary to ours. Zappacosta et al.[31] reported that persistent post-conization mRNA positivity was associated with an increased risk of recurrence or virus persistence after LEEP, and Frega et al.[32] and Persson et al.[33] similarly found E6/E7 mRNA useful for predicting recurrent disease in women treated for CIN2/3. More recently, Carcea et al.[34] and Loayza et al.[35] further supported the clinical value of mRNA testing during post-treatment follow-up, with high reported sensitivity and specificity. However, not all evidence is consistent: a systematic review by Cuschieri et al.[36] noted that some E6/E7 mRNA assays showed limited sensitivity for detecting residual disease, which aligns with our findings.

Two further considerations may explain the discrepancy beyond those discussed above. First, the definition of recurrence differed across studies—some regarded any residual/newly detected lesion (CIN1+) as recurrence, whereas others required CIN2 + ; such inconsistency directly affects recurrence rates and thus predictive estimates. Second, our very low recurrence rate (3/91, 3.3%) and the small number of events substantially limited statistical power, and because all patients had histologically confirmed negative margins, our cohort likely represents a lower-risk group in which the incremental value of mRNA testing is diminished.

This study offers several strengths. It addresses a critical clinical question by focusing on recurrence risk assessment in patients with CIN following LEEP, providing insights that may improve follow-up strategies. The use of real-world data enhances the study’s applicability, particularly within a single-center setting with standardized protocols, reflecting actual clinical practice. Rigorous inclusion and exclusion criteria ensured sample homogeneity, minimizing confounding factors, and increased internal validity. By reporting negative findings, the study contributes to a clearer understanding of the limitations of E6/E7 mRNA as a biomarker, underscoring the need for more effective markers. In addition, the multi-layered statistical approach—evaluating sensitivity, specificity, PPV, and NPV—reinforces the methodological rigor of the study.

This study also has several limitations. The sample size was relatively small, with only 3 recurrence cases, which may reduce the statistical power and generalizability of the findings, particularly with respect to recurrence prediction. As a single-center study, the sample is limited to a specific population, potentially affecting external validity; a multicenter approach could enhance generalizability. The 24-month follow-up period may also be insufficient to capture long-term recurrences, and a longer follow-up could provide a more comprehensive assessment of the role of E6/E7 mRNA in recurrence monitoring. Although strict inclusion criteria were applied, factors influencing recurrence—such as lifestyle, comorbid infections, and immune status—were not fully controlled, which could affect outcomes. The sensitivity of the E6/E7 mRNA testing method may also be limited, and more advanced technology could improve its predictive accuracy. Finally, this study focused solely on E6/E7 mRNA without comparing it with other potential biomarkers, such as p16 or Ki-67, which limits a full exploration of its performance within a multi-marker framework. Future studies with larger, multicenter cohorts and extended follow-up periods are recommended to validate these findings and further assess the clinical utility of E6/E7 mRNA in predicting recurrence in patients with CIN.

## Conclusions

This study found that HPV E6/E7 mRNA status, whether preoperative or postoperative, did not reliably predict recurrence in patients with cervical intraepithelial neoplasia (CIN) following LEEP. Although a significant reduction in mRNA positivity was observed after surgery, the low sensitivity of this biomarker limits its effectiveness as a standalone tool for recurrence monitoring. These results underscore the need for additional or alternative biomarkers with greater predictive power to enhance post-LEEP follow-up strategies. Further research with larger, multicenter cohorts and extended follow-up periods is warranted to validate these findings and explore more comprehensive approaches to recurrence prediction in patients with CIN.
